# International Congress for Study of Alcoholism

**Published:** 1889-09

**Authors:** 


THE International Congress for the study of questions relating to alcoholism was opened July 29th—Dujardin-Beaumetz in the chair.

The first question taken up was Drinking Places and the Consumption of Alcohol.

After a thorough discussion by Drysdale of England, Vaul-Leroy of Brussels, Petihan of Liege, Ivonsco of Roumania, Millet of Switzerland, Candelier (Belgium), Neiff of Switzerland, Gourand and Dubrandy of France, the following resolutions were passed:

*First.*—That the increase of the consumption of alcohol is the principal cause of insanity, of suicide, and of crime.

*Second.*—That the reducing the number of saloons is one of the means of reducing the consumption of alcohol. The Congress issues this request that the Government take the necessary measures for diminishing the number of drinking places.

The Congress then discussed The Deleterious Influence of the Abuse of Alcoholic Drinks. After discussion, the following resolutions were adopted:

*First.*—The Congress, in face of the dangers with which alcoholism menaces society, issues this request, that alcoholics, who are convicted of a violation of law, or of a crime, but who receive the benefit of a judgment of not guilty, because of their irresponsible state, shall be confined in special institutions, which shall be rather houses for treatment than houses of correction.

*Second.*—That the Congress of Paris, like the Congress held in Brussels in 1880, issues the request that chronic alcoholics having lost their power of self-control, shall be under the supervision of the public minister, arrested and placed by sentence in one of these special institutions.

The third question for consideration was Healthy Drinks for the Masses. The following resolutions were passed:

The Congress, considering that the impure alcohols are eminently toxic, and that even ethylic alcohol can be dangerous, and that it would be useful to keep under surveillance places where alcoholic drinks are sold, issues this request—

*First.*—That impure alcohols shall be prohibited, and that a very high tax shall be placed upon pure alcohol.

*Second.*—That the tax on healthy drinks shall be lowered.

*Third.*—That drinking places shall be placed under active surveillance, and that toward this end municipal laboratories shall be established.

*Fourth*—That saloons under surveillance shall be established near the laborers' quarter.

*Fifth.*—That temperance cafés shall be established.

—*Journal de Médicine de Paris*, August 4, 1889.

